# Locally adapted guidelines: a scoping review

**DOI:** 10.1186/s13643-025-02808-0

**Published:** 2025-03-21

**Authors:** Dawid Pieper, Alexander Pachanov, Carolin Bahns, Robert Prill, Christian Kopkow, Eni Shehu, Adaptation Working Group-Guidelines International Network, Yang Song, Yasser Sami Amer, Airton Tetelbom Stein, Kyung-Eun Anna Choi

**Affiliations:** 1 Brandenburg Medical School Theodor Fontane (MHB), Faculty of Health Sciences Brandenburg, Institute for Health Services and Health Systems Research, Immanuel Klinik Rüdersdorf, Seebad 82/83, Rüdersdorf Bei Berlin, 15562 Germany; 2 Evidence Based Practice in Brandenburg: A JBI Affiliated Group, University of Adelaide, Adelaide, Australia; 3 Department of Therapy Science I, Brandenburg University of Technology Cottbus-Senftenberg, Senftenberg, Germany; 4 Center of Orthopaedics and Traumatology, Brandenburg Medical School Theodor Fontane, University Hospital Brandenburg/Havel, Brandenburg an Der Havel, Faculty of Health Science Brandenburg, Brandenburg an Der Havel, Germany; 5 Brandenburg Medical School Theodor Fontane, Centre for Health Services Research, Neuruppin, Germany; 6 Health Services Research Group, Research Centre Medical Image Analysis and Artificial Intelligence, Faculty of Medicine/Dentistry, Danube Private University, Krems-Stein, Austria

**Keywords:** Clinical practice guidelines, Contextualization, Regional, Implementation, Evidence-based practice, Usability

## Abstract

**Background:**

Clinical practice guidelines (CPGs) often fail to be fully implemented in practice. One barrier to CPG implementation is inconsistency between recommendations and existing practice patterns. This can include patients, personnel, structure, availability of resources, cultural and ethical values. To account for this, it is feasible to tailor national CPGs to a regional or local context (e.g. hospital). Local ownership can be beneficial and help to implement the guideline without affecting guideline validity. This process is also known as guideline adaptation. We aimed to identify randomized controlled trials (RCTs) investigating the effectiveness of locally adapted CPGs.

**Methods:**

We performed a scoping review, following the JBI guidance. The scoping review was registered with the Open Science Framework (https://osf.io/3ed2w). The intervention had to be a locally adapted guideline (locally meaning adapted to any delineated area and/or entity at subnational and/or transnational level). Co-interventions were accepted. We did not restrict the control group. As we considered locally adapted guidelines as an intervention, and it seems feasible to test locally adapted guidelines in trials, we only considered RCTs, including cluster-RCTs. PubMed and Embase were searched in November 2024. Two reviewers independently screened titles and abstracts, full-text articles, and charted data. Conflicts were resolved by involving a third reviewer. Data were summarized descriptively. The findings were discussed with knowledge users.

**Results:**

Five cluster RCTs reported in 8 publications and published between 2000 and 2010, were included. The trials originated from the UK, Scotland, Australia, the US, and the Netherlands. The adapted CPGs focused on diabetes, asthma, smoking cessation, mental disorders, and menorrhagia and urinary incontinence. The number of sites (e.g. practices) ranged from 4 to 30. Reporting was mostly insufficient to understand how adaptation was performed. Interventions always included some form of dissemination, such as educational meetings or workshops.

**Conclusions:**

There is a lack of RCTs investigating the effectiveness of locally adapted guidelines. A systematic review is unwarranted due to the clinical and methodological heterogeneity of these trials. The identified studies were largely conducted over 20 years ago, highlighting a significant knowledge gap. The reasons for the lack of similar studies today are unclear, which is surprising given advances in adaptation frameworks in guideline development. As the importance of contextualization is emphasized, future studies on locally adapted guidelines should be conducted with strong rationale supported by local data. Without a sound rationale, there is a risk that evidence-based, high-quality guidelines could be undermined. In future trials, authors should closely adhere to reporting guidelines.

**Systematic review registration:**

https://osf.io/3ed2w.

**Supplementary Information:**

The online version contains supplementary material available at 10.1186/s13643-025-02808-0.

## Background

Clinical practice guidelines (CPGs) are critical for improving healthcare, yet adherence to their recommendations often remains suboptimal. Numerous factors contribute to the limited implementation of CPGs, which can vary by clinical field and specific recommendations [1, 2]. A key barrier is the misalignment between CPG recommendations and existing practice patterns [3]. The local context plays a pivotal role in determining whether CPGs are implemented effectively. As such, understanding the local context is essential for devising effective implementation strategies [2, 4]. Tailoring CPG recommendations to local circumstances can increase to their uptake [5]. Furthermore, local ownership can be beneficial and help with implementing the guideline, while not affecting guideline validity [3].

Local contexts can vary based on patient demographics, workforce characteristics, healthcare infrastructure, resource availability, and sociocultural and ethical considerations [6]. These differences may make interventions recommended in CPGs impractical or infeasible. This underscores the importance of contextualizing CPGs. Schünemann et al. described contextualization as the formal integration of local evidence and criteria to adapt or develop recommendations from trustworthy guidelines, ensuring their appropriateness for the target setting [6]. This process is also known as guideline adaptation, which the Guidelines International Network (G-I-N) defines as “the systematic approach to the modification of a guideline(s) produced in one cultural and organisational setting for application in a different context”. Adaptation can be either formal or informal. In contrast to informal adaptation, formal adaptation is based upon a described methodology or framework [7].

The World Health Organization (WHO) develops CPGs that need to be tailored to each country's specific needs. It is common to adapt CPGs from one country to another. Several examples of this were identified in a recent scoping review (ScR) [8]. However, national CPGs can also be adapted from a national to a regional or local level. At the local level, this may include adaptations to single hospitals. Former studies have already evaluated such adaptations [9–11]. Also, the use of guideline recommendations for deriving standard operating procedures in hospitals is possible and can be seen as a form of adaptation [12, 13].

Not only systematic reviews (SRs) but even overviews of reviews investigating implementation strategies have been published [14, 15]. Contextualization has been described as one of the most promising strategies [16]. Studies typically examine multifaceted interventions. (Cluster-) Randomized controlled trials (RCTs) investigating the impact on implementation and patient outcomes have been conducted and found to be feasible. According to our experience in developing CPGs, the idea of contextualization is often neglected or not considered at all. To the best of our knowledge, there is no evidence synthesis focusing on locally adapted guidelines. Thus, we set out to perform a ScR on studies investigating the effectiveness of locally adapted CPGs.

## Methods

We followed the JBI (formerly Joanna Briggs Institute) guidance to prepare the protocol for our ScR [17]. We published the protocol a priori on the Open Science Framework (https://osf.io/3ed2w). Equally, we were informed by the updated JBI guidance for conducting ScRs [18]. When reporting our results we adhered to the Preferred Reporting Items for Systematic Reviews and Meta-analysis Extension for Scoping Reviews (PRISMA-ScR) [19]. Deviations from the protocol are reported providing a rationale in the corresponding section where deviations occurred. We also followed the suggestions from the text recycling project [20].

The specific objectives of this ScR were:

1. Identify the available evidence studies investigating the effectiveness of locally adapted CPGs.
2. Examine the study design features.
3. Investigate how adaptation was performed.
4. Identify and analyze potential knowledge gaps.
5. Inform the conduct of a subsequent SR.

### Eligibility criteria

- Locally adapted guideline, with co-interventions allowed
- (Cluster-)RCTs
- Published in English or German

We included studies, if they met the following criteria. The intervention must be a locally adapted guideline. According to the Institute of Medicine, ‘clinical guidelines are statements that include recommendations intended to optimize patient care that are informed by a systematic review of evidence and an assessment of the benefits and harms of alternative care options’ [21].

We emphasize that the term ‘locally’ lacks a clear and universally accepted definition. In the context of guideline adaptation ‘local adaptation’ often refers to adapting a guideline from one country to another [8]. For the purpose of our ScR, we defined 'local' as any specific area or entity at either subnational (e.g., federal state, hospital) or transnational levels, while adaptation refers to modifying a CPG so that it suits to a given context. This definition was informed by the existence of locally adapted guidelines at these levels [9–11]. Studies that adapted not the entire CPG but only specific parts, such as chapters or individual recommendations, were also eligible. Since implementation strategies for CPGs are often designed as multifaceted interventions, we permitted co-interventions. This could also include implementation strategies for the locally adapted guideline itself. Locally adapted guidelines can be evaluated for their effectiveness in the same way as non-adapted guidelines, and the best available evidence for testing interventions comes from (cluster-) RCTs; therefore, we focused exclusively on this study design and excluded others. Additionally, we did not impose any restrictions on the control group and the outcomes measured. Due to resource constraints, we considered only publications in English or German. We decided post hoc that excluding articles due to language would only be imposed at full-text level [22].

### Information sources and search

We searched the following databases from inception: PubMed and EMBASE (Embase.com). The search strategies were developed by DP, who has experience as an information specialist, and checked by another member (AP) of the team against the Peer Review of Electronic Search Strategies (PRESS) criteria [23]. Following the idea of an objective approach [24, 25], we used potentially eligible studies [26–28] known to us prior to executing the ScR process. The search strategy consisted of terms related to guidelines, adaptation and geographical patterns. In addition, we applied the Cochrane RCT sensitivity maximising filter for PubMed [29]. As a next step the PubMed search strategy was translated for Embase (Embase.com). The initial searches in both databases were conducted on 14 November 2023 and updated on 22 November 2024. The final search strategies can be found in supplementary file 1.

On 19 February 2024, we performed forward and backward citation tracking using the Citationchaser Shiny app [30], and updated the forward citation tracking on 22 November 2024. We also contacted the authors of the included studies. Furthermore, we reached out to the G-I-N adaptation working group. The G-I-N adaptation working group contacted all group members for further potential studies following a project presentation in July 2024 (see also the section on knowledge user involvement).

### Study selection

Records were uploaded to the reference management tool Endnote. Two reviewers (CB, ES) screened independently titles and abstracts using Rayyan [31]. No piloting was performed. All reports (full-texts) deemed potentially relevant were retrieved. Again, each report was independently screened by two reviewers (CK, AC). Reasons for exclusion were recorded. At any stage, disagreements between the reviewers were resolved through discussion or by involving an additional reviewer (DP). In the case of missing relevant information, we planned to reach out to the study authors.

### Data extraction

Two members of the team (RP, HH) independently extracted relevant data from each included article. Again, disagreements between the reviewers were resolved through discussion or by involving an additional reviewer (DP). Multiple reports of a single study were combined and checked for consistency. We extracted the following data items:

- First author.
- Initiator(s).
- Year.
- Country.
- Geographical area or entity to which the guideline was adapted.
- Source(s) and date of publication of original guideline(s).
- Rationale for adaptation (e. g. local evidence).
- Method/transparency of adaptation.
- Adapted guideline.
- Patient population(s).
- Sample size
- Service provider(s) setting.
- Study design.
- Study period.
- Effect measures.
- Funding.
- Reported COI, memberships, organizations.
- Availability of a study protocol (as reported by the study authors).

We developed a data extraction sheet based on these items. As we expected to include less than 10 studies, we decided not to pilot test the data extraction form. However, the extracted data sheets once completed were shared with the team and discussions took place whether amendments to the data extraction form were necessary.

### Critical appraisal

We did not perform any critical appraisal of the included studies.

### Synthesis

Characteristics were analyzed descriptively using frequencies and percentages. We presented the number of studies according to the year of publication, country, geographical area to which the guideline was adapted, adaptation method, and sample size, study design, and type of data source. We also characterized the types of included studies with respect to their methodological characteristics. Given the number of included studies, we decided to present our results in tables. We highlighted differences between those studies.

### Knowledge user involvement

Some of us were or are actively involved in guideline development (CB, CK, DP, RP). Furthermore, we reached out to discuss our results with experts from 1) the German Association of the Scientific Medical Societies (AWMF), 2) German Agency for Quality in Medicine (ÄZQ) and 3) German Guideline Program in Oncology. The online meeting took place in December 2024. Furthermore, we were given the opportunity to present our results in a G-I-N adaptation working group meeting in July 2024. The latter was not planned before but arose during contacting the working group. We did not plan to involve any patients or aimed for any other form of public involvement.

## Results

**Fig. 1:**
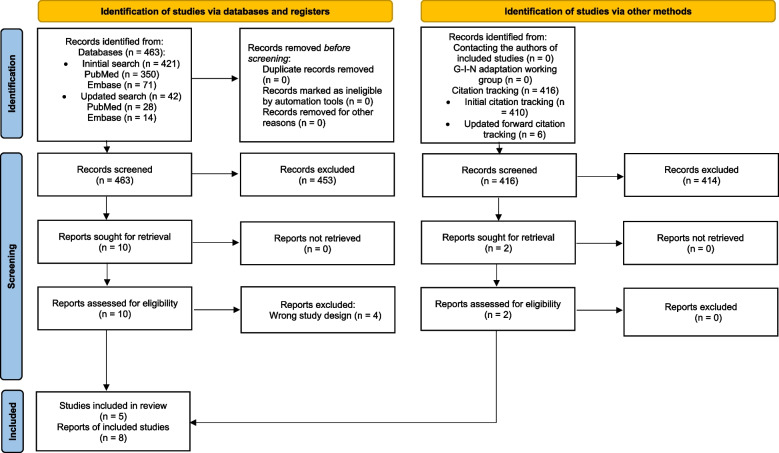
Fliow diagram

The initial and updated database searches yielded a total of 463 records: 378 from PubMed and 85 from Embase. No duplicates were identified, either manually or using Rayyan. After title and abstract screening, 453 records were excluded, leaving 10 for full-text screening. Ultimately, we included five studies [10, 26, 28, 32, 33]. For one study, we identified two reports [26, 34]. Citation tracking of the included reports identified 416 additional records, of which 414 were excluded after title and abstract screening. The remaining two records [35, 36] were assessed in full-text, and were included as additional reports of a study identified through the database search [28]. No additional records or reports were identified through other sources. The selection process is outlined in the PRISMA flow diagram (Fig. 1).

### Study characteristics

The included studies were from five different countries (Australia, the Netherlands, England, Scotland, United States). Four studies were restricted to a pre-defined region/area, while one study [10] was conducted across the whole country (Tables 1 and 2). Study periods were only reported for three studies and spanned totally from 1997 to 2002. The two studies not reporting their study period were published in 2000 and 2008 [10, 28], respectively. All studies were (Cluster-)RCTs. In four studies, randomization was performed at the level of practices. One practice could include more than one practitioner. One study [10] applied a cross-over design. This study tested the same intervention for two different conditions (menorrhagia or urinary incontinence). Hospitals were randomized to one condition, while serving as a control for the second condition. No study mentioned a protocol having been published before the trial. All studies receiving funding and authors declared to have no conflicts of interest.

**Table 1:** Study characteristics

Sudy	Study period	Country	Area/entity to which the guideline was adapted	Original guideline	Adapted guideline	Initiator and rationale	Adaptation method
Liaw 2008, Sulaiman 2010	2001	Australia	Melbourne	paediatric asthma guidelines (not specified further)	nr	Nr	Group discussions
Van Bruggen 2008	nr	Netherlands	Apeldoorn area	Diabetes mellitus type 2 CPG of the Dutch College of General Practitioners	Modifications to cut offs; de novo rules for referral back	Insurance company, rationale nr	Nr
Yano 2008	2000–2002	United States	5 states in the south west	U.S. Public Health Service smoking cessation guidelines (not specified further)	nr	Nr	Nr
Croudace 2003	1997–1999	England	Bristol	WHO ICD-10 PHC Guidelines for Diagnosis and Management of Mental Disorders (1996)	‘The Bristol version’ of the original guideline	Nr, to engender shared ownership between primary and secondary care practitioners	workshops based on a modified nominal group technique
Chadha 2000	nr	Scotland	East/west	National condition specific guidelines (not specified further)	nr	nr	nr

*Nr* not reported, *WHO* World Health Organization, *PHC* Primary Health Care

**Table 2:** Intervention characteristics

Study	Patient population	Interventions, n patients	service provider(s)setting, n	Level of outcome measurement	Effect measures
Liaw 2008, Sulaiman 2010	asthma	Group 1: Educational workshops + LAG (n nr) Group 2: LAG (n nr) Group 3: alternative educational program (n nr)	Group 1: 10 Practices (18 GPs) Group 2: 9 Practices (18GPs) Group 3: 10 Practices (15 GPs)	GPs only	process of care knowledge confidence
Van Bruggen 2008	Diabetes mellitus type 2	Group 1: usual care (*n* = 818 randomized) Group 2: LAG (*n* = 822 randomized)	General practices, *n* = 30 (in total)	Patients	Clinical (eg. HbA1c) Quality of life satisfaction
Yano 2008	Smoking cessation	Group 1: quality improvement program (consisting of educational materials, structured evidence reviews, LAG, local priority setting, QI plan development and adaptation, audit & feedback, expert review, local opinion leaders (*n* = 925 enrolled) group 2: Guideline (not specified); audit & feedback (*n* = 1,016)	VA Health Care Facilities; Practices, *n* = 9 in each group	Patients	Attendance rate smoking cessation
Croudace 2003	Mental disorders	Group 1: LAG Group 2. usual care *N* = 2,328 (totally evaluated patients)	Group 1: 15 Practices (56 GPs) Group 2: 15 Practices (60 GPs)	Patients and practices	diagnosis Health status
Chadha 2000	Menorrhagia or urinary incontinence (dependent on group assignment)	LAG *N* = 888 in total	Hospitals (*n* = 4)	patients	Process of care Health status

*VA* Veteran Affairs, *GP* general practitioners, *LAG* locally adapted guideline, *QI* quality indicators

### Adaptation characteristics

All studies named the original guideline that underwent adaptation, while references were provided in only two studies (Table 1). Van Bruggen et al. specified the original guideline that underwent adaptation [37]. Changes made largely focused on different cut-offs (, e.g., recommended blood pressure changed from < 150/85 to < 140/85), but also included de novo recommendations including explicit rules for referral back to primary care that were not included in the original guideline at all. Croudace et al. also specified the original guideline [38] without clarifying what changes were made. However, they provided a reference to the adapted guideline. For the other three studies neither the adapted guideline nor the changes made were mentioned. The adaptation process was informal in two studies. Liaw et al. 2008 referred to group discussions [26], while Croudace et al. performed workshops based on a modified nominal group technique [32]. No information was reported about the adaptation methodology for the other three studies. Only one study specified its aim beyond improving health care (outcomes) [32]. One study explicitly mentioned the rationale for adaptation being ‘to engender shared ownership between primary and secondary care practitioners’ [32]. No study reported having used local data to support their rationale. Only one study specified who initiated the guideline adaptation process. In the study from the Netherlands the initiator was an insurance company, while no rationale was provided [28].

### Intervention and outcome characteristics

The included studies dealt with different topics such as asthma [26], diabetes mellitus type 2 [28], smoking cessation [33], mental disorders [32], and menorrhagia or urinary incontinence [10]. Four studies comprised two study groups, while one study had three study groups [26]. Locally adapted guidelines were the sole intervention in four studies [10, 26, 28, 32]. Locally adapted guidelines were part of multifaceted interventions in two studies [26, 33]. Practices were allocated to the interventions in four studies, while allocation took place at the hospital level in one study [10]. Within the four studies, three studies randomized between 29 to 30 practices [26, 28, 32]. The number of patients enrolled or analyzed ranged approximately between 1,000 and 2,000 patients. One study did not analyze outcomes at patient level, but only at practitioner level [26]. Three studies focused on patients outcomes only [10, 28, 33], while one study considered both [32], patient outcomes and practitioner outcomes. Health status or quality of life was most frequently measured (*n* = 3 studies) at patient level. In both studies investigating outcomes at practitioner level indicators of process of care, including adherence with guideline recommendations, were measured.

## Discussion

Our ScR on effectiveness studies investigating locally adapted guidelines found only five studies indicating that there is not much research on it. In addition, the studies are quite old and reporting prohibits from a better understanding what is in particular true for the adaptation processes. Given that there has been much advance in guideline adaptation methodology after the studies have been published, our ScR reveals an important research gap.

We were only able to identify five RCTs from multiple countries investigating locally adapted guidelines. These studies were published between 2000 and 2008. Considering the time to publish the study results, all studies have been conducted more than 20 years ago. This is surprising for two major reasons: Firstly, the importance of the local context or contextualization has been repeatedly emphasized over the last couple of years [39, 40]. Context is shaped by a combination of environmental conditions, organizational structures, cultural norms, and external influences. The success of interventions is deeply tied to the environment in which they are implemented, as the unique characteristics of the setting influence both the process and outcomes of implementation. Implementation science has been dealing with this for a long time [40], also in relation to CPGs [5, 14, 41, 42]. It has also become frequent for interventions being adapted to their local context [43]. We acknowledge that contextualization in the context of CPGs mostly refers to the national level being considered the context [8]. Context itself is not well conceptualized [44]. Although we came up with a definition for our ScR for the sake of operationalization, we concede that local contexts are not equal to national or geographical borders. Secondly, there has been much methodological development in guideline adaptation mostly after the included studies were conducted. A review identified eight existing adaptation frameworks in 2017 [45]. According to this review, the first adaptation framework was developed by the Royal College of Nursing in 2000 [46]. The second oldest framework is ADAPTE, first published in 2005 and updated in 2009 [47]. Thus, our finding that most authors did not use a formal adaptation framework is obvious, as only one study could have potentially applied one [28]. In addition, an analysis of adapted CPGs showed that only 40% did use a formal adaptation method [48]. However, only CPGs published until 2015 were considered. Given the amount of adaptation frameworks available and the increasing experience with it, the proportion of CPGs using a formal method when undergoing adaptation seems very likely.

It is also important to note that the reporting in the included studies was suboptimal. This is in particular true for the adaptation process. Again, this can be explained by the fact that adaptation methodology was not well developed when the studies were conducted. Only one study made the changes to the original guideline explicit [28]. Equally, the interventions were not well described, making replication not possible. Although the first CONSORT (Consolidated Standards of Reporting Trials) statement was published in 1996, and thus could have been followed by the authors, only the updated 2010 version was adhered to by many journals [49]. We observed suboptimal reporting in particular for the description of the interventions, where reporting guidelines such as TIDieR (template for intervention description and replication) exist [50], but have only become available after the studies have been completed.

We only considered RCTs for eligibility in our ScR. Given the low number of finally included studies this choice might be criticized. However, we argue that RCTs are the best study design for conducting such studies. It might be debated whether the included studies should be labelled either as effectiveness studies or implementation studies. It is important to stress that randomization is also considered to be the gold standard in implementation science [51, 52]. We also observed different types of outcomes measured. Collecting patient outcomes only does more resemble the idea of effectiveness trials, while collecting data at the provider level does more align with the idea of implementation trials, although effectiveness-implementation hybrid designs also exist [53]. Considering other study designs for eligibility in our ScR would likely not result in a huge number of additional studies as informed by a quick focused search in PubMed. Interestingly, other non-randomized studies [9, 11, 27] known to us are from the same time period as our included studies.

It can be further questioned whether the small number of included studies should be interpreted in a way that locally adapted guidelines (as defined by us) are not being investigated, or that the number of locally adapted guidelines is very low. With an increasing number of adaptation frameworks, the number of locally adapted guidelines could rise, along with the number of RCTs investigating them. Some studies have adapted national guidelines to single hospitals [9, 11]. It seems sensible that CPG recommendations could also serve as a basis for developing standard operating procedures [12, 13], although this is a very unstudied research field as most standard operating procedures implemented in hospitals are not freely accessible making it hard to understand how they were developed.

### Limitations

Our ScR has some limitations. We opted not to use the standard PCC (population, concept, context) mnemonic for ScRs, as it did not align well with our research question. Although we included only articles in English and German, and thus might have missed potentially relevant articles, we did not exclude articles based on language during the abstract screening process. Given the topic, it is possible that relevant papers are being published in national languages and in journals that are primarily distributed locally or regionally. However, this concern might have been more significant had we focused on multiple study designs, as RCTs are primarily published in international journals. To address our limitations regarding language and database coverage, we conducted forward and backward citation tracking to identify additional potentially relevant literature.

## Conclusion

There is a paucity of RCTs investigating the effectiveness of locally adapted guidelines. A SR of their effectiveness is currently unwarranted due to the clinical and methodological heterogeneity of these trials. The identified studies were largely conducted more than 20 years ago, highlighting a significant knowledge gap. The reasons for the lack of similar studies being conducted today are not obvious. This is particularly surprising given the advances in developing and using adaptation frameworks in guideline development. As the importance of contextualization is repeatedly emphasized, studies investigating locally adapted guidelines should be conducted in the future. However, there must be a strong rationale for why local adaptations are needed, which can be supported by the use of local data. Without a sound rationale for local adaptations, there is a risk that evidence-based, high-quality guidelines could be undermined. If done correctly and implemented successfully, locally adapting guidelines might have the potential to improve health outcomes. When conducting future trials, authors should closely adhere to reporting guidelines.

## Supplementary Information

Supplementary Material 1.

Supplementary Material 2.

## Data Availability

All data generated or analyzed during this study are included in this published article.

## Abbreviations

AWMF: German Association of the Scientific Medical Societies
ÄZQ: German Agency for Quality in Medicine
CONSORT: Consolidated Standards of Reporting Trials
CPG: Clinical practice guideline
G-I-N: Guidelines international network
PCC: Population, concept, context
PRESS: Peer Review of Electronic Search Strategies
PRISMA-ScR: Preferred Reporting Items for Systematic Reviews and Meta-Analyses Extension for Scoping Reviews
RCT: Randomized controlled trial
ScR: Scoping Review
SRs: Systematic Reviews
TIDieR: Template for intervention description and replication
WHO: World Health Organization
