# Supplementary material for: Locally adapted guidelines: a scoping review
Source: Syst Rev. 2025 Mar 21;14:66. doi: 10.1186/s13643-025-02808-0 (PMC11927138; doi:10.1186/s13643-025-02808-0)
Supplement: Supplementary file 1 — Supplementary Material 1. [file 13643_2025_2808_MOESM1_ESM.docx]

**Supplementary file 1**

**Electronic search terms and syntax**

**PubMed**

("Practice Guidelines as Topic"[MeSH Terms] OR "guideline*"[Title]) AND "adapt*"[Title/Abstract] AND ("local*"[Title/Abstract] OR "regional*"[Title/Abstract] OR "context*"[Title/Abstract]) AND ("randomized controlled trial"[Publication Type] OR "controlled clinical trial"[Publication Type] OR "randomized"[Title/Abstract] OR "placebo"[Title/Abstract] OR "drug therapy"[MeSH Subheading] OR ("randomly"[Title/Abstract] OR "trial"[Title/Abstract] OR "groups"[Title/Abstract])) NOT ("animals"[MeSH Terms] NOT "humans"[MeSH Terms])

Date of initial search: 14.11.2023
No. of hits: 350

Date of updated search: 22.11.2024
No. of hits: 28

**Embase (Embase.com)**

('practice guideline'/exp OR guideline*:ti) AND adapt:ti,ab AND (local*:ti,ab OR regional:ti,ab OR context*:ti,ab) AND ('randomized controlled trial'/exp OR 'controlled clinical trial'/de OR random*:ti,ab,tt OR 'randomization'/de OR 'intermethod comparison'/de OR placebo:ti,ab,tt OR compare:ti,tt OR compared:ti,tt OR comparison:ti,tt OR ((evaluated:ab OR evaluate:ab OR evaluating:ab OR assessed:ab OR assess:ab) AND (compare:ab OR compared:ab OR comparing:ab OR comparison:ab)) OR ((open NEXT/1 label):ti,ab,tt) OR (((double OR single OR doubly OR singly) NEXT/1 (blind OR blinded OR blindly)):ti,ab,tt) OR 'double blind procedure'/de OR ((parallel NEXT/1 group*):ti,ab,tt) OR crossover:ti,ab,tt OR 'cross over':ti,ab,tt OR (((assign* OR match OR matched OR allocation) NEAR/6 (alternate OR group OR groups OR intervention OR interventions OR patient OR patients OR subject OR subjects OR participant OR participants)):ti,ab,tt) OR assigned:ti,ab,tt OR allocated:ti,ab,tt OR ((controlled NEAR/8 (study OR design OR trial)):ti,ab,tt) OR volunteer:ti,ab,tt OR volunteers:ti,ab,tt OR 'human experiment'/de OR trial:ti,tt) NOT ((((random* NEXT/1 sampl* NEAR/8 ('cross section*' OR questionnaire* OR survey OR surveys OR database OR databases)):ti,ab,tt) NOT ('comparative study'/de OR 'controlled study'/de OR 'randomised controlled':ti,ab,tt OR 'randomized controlled':ti,ab,tt OR 'randomly assigned':ti,ab,tt) OR ('cross-sectional study'/de NOT ('randomized controlled trial'/exp OR 'controlled clinical trial'/de OR 'controlled study'/de OR 'randomised controlled':ti,ab,tt OR 'randomized controlled':ti,ab,tt OR 'control group':ti,ab,tt OR 'control groups':ti,ab,tt)) OR ('case control*':ti,ab,tt AND random*:ti,ab,tt NOT ('randomised controlled':ti,ab,tt OR 'randomized controlled':ti,ab,tt)) OR ('systematic review':ti,tt NOT (trial:ti,tt OR study:ti,tt)) OR (nonrandom*:ti,ab,tt NOT random*:ti,ab,tt) OR 'random field*':ti,ab,tt OR (('random cluster' NEAR/4 sampl*):ti,ab,tt) OR (review:ab AND review:it)) NOT trial:ti,tt OR ('we searched':ab AND (review:ti,tt OR review:it)) OR 'update review':ab OR ((databases NEAR/5 searched):ab) OR ((rat:ti,tt OR rats:ti,tt OR mouse:ti,tt OR mice:ti,tt OR swine:ti,tt OR porcine:ti,tt OR murine:ti,tt OR sheep:ti,tt OR lambs:ti,tt OR pigs:ti,tt OR piglets:ti,tt OR rabbit:ti,tt OR rabbits:ti,tt OR cat:ti,tt OR cats:ti,tt OR dog:ti,tt OR dogs:ti,tt OR cattle:ti,tt OR bovine:ti,tt OR monkey:ti,tt OR monkeys:ti,tt OR trout:ti,tt OR marmoset*:ti,tt) AND 'animal experiment'/de) OR ('animal experiment'/de NOT ('human experiment'/de OR 'human'/de))) AND [embase]/lim NOT [medline]/lim NOT [pubmed-not-medline]/lim

Date of initial search: 14.11.2023
No. of hits: 71

Date of updated search: 22.11.2024
No. of hits: 14
